# Supplementary material for: Genetic substructure and complex demographic history of South African Bantu speakers
Source: Nat Commun. 2021 Apr 7;12:2080. doi: 10.1038/s41467-021-22207-y (PMC8027885; doi:10.1038/s41467-021-22207-y)
Supplement: Supplementary file 1 — Supplementary Information [file 41467_2021_22207_MOESM1_ESM.pdf]

Genetic substructure and complex demographic history of South African Bantu speakers

Dhriti Sengupta[1#], Ananyo Choudhury[1#], Cesar Fortes-Lima[2], Shaun Aron[1], Gavin Whitelaw[3,4], Koen Bostoen[5], Hilde Gunnink[5], Natalia Chousou-Polydouri[6], Peter Delius[7], Stephen Tollman[8], F Xavier Gómez-Olivé[8], Shane Norris[9], Felistas Mashinya[10], Marianne Alberts[10], AWI-Gen Study[*], H3Africa Consortium[*], Scott Hazelhurst[1,11], Carina M. Schlebusch[2,12,13,&] and Michèle Ramsay[1,14,&].

SUPPLEMENTARY NOTES

Note 1. Linguistic phylogeny of the South Eastern Bantu languages

In order to compare the genetic relatedness of the populations included in this study with their linguistic affiliation, we include in this paper a new comprehensive linguistic phylogeny of the South-East Bantu (SEB) languages of South Africa, which is part of a larger phylogenetic study of Southern African Bantu languages (Gunnink et al, in prep). The phylogeny is based on lexical data for 100 concepts in 69 Bantu language varieties, 34 of them part of SEB (20 of which are spoken in South Africa) and 35 outgroup languages belonging to different major Bantu branches[1]. The lexical data were organized in 1304 partial cognate sets (form- meaning associations) and coded as binary characters. The resulting matrix was analyzed with Bayesian inference methods as implemented in MrBayes (v3.2.7)[2,3] using a restriction-site model[4]. The full majority-rule consensus tree is shown in **Supplementary Fig. 3b**.

The new lexical linguistic phylogeny proposes that SEB languages are situated within the Eastern branch of Bantu[1]. They descend from a most recent common ancestor that is distinct from other Eastern Bantu languages. Their closest relatives are spoken to the northeast of their distribution area.

When it comes to internal relationships between SEB languages, the linguistic phylogeny supports the well-known split between the groups known as Sotho (including Pedi, Sotho, and Tswana and several smaller varieties not recognized as official) and Nguni (including Zulu, Swazi, Xhosa, Ndebele, and several smaller varieties without official status). However, it also shows that Nguni is closer to Tsonga than to Sotho. Tsonga and its closest relatives in Mozambique form a clade sister to Nguni. Although Tsonga is usually seen as an outgroup, it is not an independent linguistic entity according to our phylogeny. It is more closely related to the languages of the Nguni group than to any other South African Bantu language. Together, Tsonga and Nguni constitute a clade that is sister to Sotho. Venda is sister to the clade uniting Sotho with Tsonga and Nguni, which is in line with the language's traditional conception as a relative outsider among South African Bantu languages.

The linguistic phylogeny is mostly in line with findings from genetics and archaeology, but there are also some interesting discrepancies. It suggests that Venda is the first language to split off from the clade clustering together all South African Bantu languages. By contrast, a well-established archaeological sequence puts the origin of Venda in interaction between Sotho and Shona speakers in the AD 1500s and 1600s.[5] Similarly, while archaeological evidence points to different episodes of expansion of agriculturalist communities into South Africa,[6] the linguistic phylogeny indicates that all present-day South African Bantu languages descend from a most recent common ancestor language that was spoken in a homeland area situated in the borderland between Zimbabwe, Mozambique and South Africa according to the principle of highest diversity within SEB. It rather supports a scenario of divergence subsequent to arrival in South Africa. Furthermore, although the identification of genetic Khoe-San admixture in certain SEB groups is mirrored in linguistic Khoe-San influence, e.g. in Xhosa and Zulu, other SEB groups show relatively high degrees of Khoe-San admixture but do no manifest linguistic Khoe-San influence, e.g. Tswana[7].

Note 2. Methods for haplotype-based clustering and Khoe-San admixture dating in SEB groups

To investigate fine-grained population sub-structure among SEB groups, we performed haplotype-based clustering analysis[8]. Phased data of SEB individuals included in the AWI-S4 dataset (**Supplementary Table 1**) were analysed using ChromoPainter/fineSTRUCTURE v4.1.1[8]. This approach models LD using the Li and Stephens's model[9] to "paint" phased chromosomes of a given recipient individual with the haplotypes of all other individuals in the dataset. We first used four randomly selected chromosomes, to estimate the following parameters after ten expectation-maximization (EM) iterations: effective population size (Ne = 158.037) and mutation rate (θ or mu=0.000308742). We then performed ChromoPainter/fineSTRUCTURE for all the autosomal chromosomes using default settings. Lastly, we plotted the MCMC pairwise-coincidence matrix and tree obtained with fineSTRUCTURE using Finegui v0.1 (available at http://www.paintmychromosomes.com), and we performed haplotype-based PCA on the basis of the co-ancestry matrix with the *eigen* R function.

To reconstruct the timeframe of admixture events between the major ancestry components in SEB populations, we used three admixture dating methods. The first method used was fastGLOBETROTTER, the recent implementation of GLOBETROTTER[10]. Briefly, fastGLOBETROTTER tests for evidence of one, two or more pulses of admixture events between two or more ancestral groups, and dates these admixture events to infer the genetic make-up of the studied admixed groups. To do so, we estimated the amount of an individual's genome that is shared with each other individual in the dataset using the chromosome painting approach implemented in ChromoPainter (v4)[8]. This approach applies the model initially introduced by (ref. [9]) to "paint" haplotypically phased chromosomes of a given recipient individual with the haplotypes of all other individuals from other populations included in the dataset. For this analysis, we selected African populations from different African regions and one European population (CEU)[11–13]. Those populations were used as both surrogate and donor populations, and SEB populations as target/recipient populations after randomly down-sampling each SEB population to 30 individuals (except for Venda, in which 24 EC samples were analysed). To estimate program's parameters such as effective population size (Ne) and mutation rate (θ), we used ChromoPainter with 10 Expectation-Maximisation (E-M) steps repeating this separately for four chromosomes (1, 6, 12, and 18) and weight-averaging the Ne and θ from the final E-M step across the four chromosomes. The estimated values were Ne=522.81 and θ=0.00126611, which were used as parameters for "painting" all chromosomes. After chromosomal painting, we used fastGLOBETROTTER to estimate admixture dates for each SEB population following recommendations from (ref. [10]). Confidence intervals (95% CI) of estimates of dates and ancestry proportions were based on 50 bootstrap replicates of the fastGLOBETROTTER procedure.

Second, we used MALDER (v1.0)[14] to test whether a SEB group is admixed between two parental sources (Khoe-San and Bantu-speaker (BS) populations) and estimate the time since admixture based on linkage disequilibrium (LD) decay with distance. All possible triplets of populations in the dataset were tested. To ensure that the varying degree of ancestral components within SEB groups and the difference in sample sizes does not affect the admixture dating, we initially randomly selected a maximum of 100 samples per SEB group in triplicate and ran the analysis. We tested for specific admixture events between each group and Khoe-San hunter-gatherer groups presented in (ref. [13]). The minimum genetic distance to start curve-fitting was set to 0.005 cM to account for short range LD between African populations. Significant results were assessed based on the amplitude of the fitted LD curves and the corresponding z-scores. Concordant results between the 3 analyses were reported. To

further test the robustness of the admixture dating, the analysis was repeated with the ethno-linguistically concordant (EC) participants only and yielded similar results.

Third, we used MOSAIC v1.3.7[15], which exploits admixture LD information to decompose the haploid genome into putative ancestry segments. For each SEB group, we modelled two- and three-way admixture models from unknown ancestral source groups, where the target population is a mosaic of segments from the donor population(s) using a two-layer Hidden Markov Model (HMM) algorithm that allowed for linkage along the haploid genome[15]. This approach can be viewed as a combination of HapMix[16] and GLOBETROTTER[10]. Based on the coancestry plots across individuals in each SEB group and averaged in each group, the best-fitting model was two-way admixture models (with the lowest expected r-squared in all the groups).

To convert the estimated admixture dates from generations to years (on Common Era, CE), we used the formula y= 1950-29*(g+1), where y is the year of admixture, g the estimated number of generations, and taking 29 years as the generation time[17]. The tested admixture models using fastGLOBETROTTER's best-guess conclusion was one date of admixture event in all SEB groups, which is consistent with the ancestry decay curves estimated using MOSAIC on the basis of two-way admixture events in SEB groups. The estimated admixture dates correlated between these two methods (**Supplementary Figure 7b**), except for the Venda group that has more variation in the admixture patterns among the individuals of this group (see SD in **Supplementary Table 3**). In both methods, the estimated source population for the Bantu-related ancestry was Baganda from Uganda[11], and for the Khoe-San-related ancestry were Southern Khoe-San groups, Karretjie and Khomani[13] (**Supplementary Table 3**). Despite similarities in admixture dates, the best Khoe-San proxy populations detected by MALDER were different from the Khoe-San proxy detected by the other two methods (**Supplementary Table 3**). In addition, estimated admixture proportions (BS range: 72-91% and Khoe-San range: 9-28%) agree with our previous results using admixture inference methods (**Supplementary Table 3**).

Note 3. Sex-specific admixture patterns

Several recent studies based on surveys of mitochondrial DNA (mtDNA) and Y-chromosome (Y-chr) haplogroups in Southern African populations have demonstrated a clear sex-biased gene flow between the Khoe-San and BS[18–20]. Among the five Y-haplogroups found to be common among the SEB of this study, three are associated with Bantu-speakers (E1b1/E-P2, E2b/E-M52, and B2a1/B-M109) and two are associated with Khoe-San populations (B2b/B-P6 and A1b1b2a), which are only 5.1% of the samples (**Supplementary Table 5, Fig. 3a**). Quality of assignment was measured using the F1 score —all assignments of E haplogroups were done with F1 score >0.89, and assignments of B haplogroups were done with F1 score >0.77. The assignment of our samples to A1b was with F1=1, though finer-scale resolution to A1b1b2a was only done with F1 in [0.60, 6.69]. The classification of the relatively few individuals with Y-haplogroups usually not associated with Africans included haplogroups assigned with F1>0.9 except for about a dozen individuals classified in J2a1a with F1<0.6.

In contrast, among the mtDNA-haplogroups detected in our dataset, the proportion of the two Khoe-San associated mtDNA-haplogroups (L0d and L0k) is about 20.5%, confirming Khoe-San biased maternal gene flow (**Supplementary Table 6, Fig. 3a**). MtDNA classification was more complicated than for Y-haplogroups due to technical limitations of the H3A custom array. Nonetheless, this array allowed high resolution and accurate calling of L0 haplogroups associated with Khoe-San ancestry/speakers (such as L0d and L0k), and could distinguish between three sub-haplogroups of L0d (L0d1, L0d2, and L0d3). However, the base of the array was from existing Illumina bead pools which has good coverage of non-African haplogroups (viz, M and N and below) and some coverage of African

haplogroups. As part of the design process, additional probes were added (Botha et al. in prep). However, the underlying array technology probes for SNPs that are within 100 bp of each other may interfere with each other (and more so as they get closer to each other). As the mitochondrial genome is too short (over 16K SNPs) and there were over 200 SNPs genotyped, the array has limitations for the coverage of other African mtDNA-haplogroups. Besides, the classifications that were made were done with reasonable quality scores (except for L2a1 with a score of 0.63), but in some cases it was at a very coarse resolution. For example, 16% of the samples were classified as L0a'b'g but could not be classified more deeply and about 5% were classified as L1'2'3'4'5'6 but could not be classified more deeply. Additional SNPs covering L0a and L0g seem to be the most pressing, and with extra coverage of L3, L4, L6 and especially L2 being desirable.

The comparison of autosomal and X-chromosome contributions in various SEB groups reiterated the overall trend of female-driven gene flow from Khoe-San (**Fig. 3b**). However, as seen with uniparental haplogroup comparisons, the degree of this sex-bias was found to vary widely between the SEB groups, with groups such as Tsonga and Zulu showing a weaker sex-bias in comparison to Xhosa and Sotho (**Fig. 3b**). Sotho shows significantly higher sex-bias in comparison to all the other SEB groups **(Supplementary Table 7).** Moreover, while some groups with higher overall Khoe-San ancestry (Tswana) demonstrate stronger sex-bias in admixture compared to groups with lower Khoe-San ancestry (Pedi), the observed variations in the level of sex-biased admixture are not driven by the differences in Khoe-San ancestry. For example, Zulu in spite of having much higher Khoe-San ancestry in comparison to the Tsonga show comparable sex-biased admixture (P-value=0.3). Although our results from both uniparental markers and admixture difference ratio overall support the existing hypothesis of sex-biased admixture between Bantu-speaking males and autochthonous Khoe-San females, the extent of this sex-biased admixture might have varied among SEB groups possibly due to various demographic and cultural factors.

Note 4. Levels of relatedness

High levels of relatedness among individuals could potentially influence PCA, admixture profiles and other population-based estimates and need to be accounted for in genome-wide association studies. The assessment of background relatedness in a dataset is, therefore, important for ensuring the robustness of various genetic inferences. Identity-by-descent (IBD) between pairs of individuals from each study site was estimated using PLINK (v1.9)[21]. Pairs of individuals with PIHAT >0.18 were considered to be highly related (equivalent to third degree and closer relationships), while individuals with PIHAT values between 0.05 and 0.18 were considered to show cryptic relatedness (between third degree and fifth degree relatives).

In the AWI-Gen dataset, we estimated that about ~36% of Tsonga participants (predominantly from the Agincourt (AGT)) and ~25% of the Pedi participants (predominantly from the Dikgale (DKG) show a very high relatedness (PIHAT >0.18) (**Supplementary Fig. 10a**). We observed a very similar trend for the number of individuals distantly related to each other (0.05<PIHAT<0.18), where Tsonga and Pedi once again have the highest numbers from AGT and DKG, respectively (**Supplementary Fig. 10b**). To investigate if these observations were biased by the unequal sample size in Tsonga and Pedi, we performed a bootstrap approach by resampling up to 100 samples for 100 iterations, and estimated the percentage of samples related within the range of 0.05<PIHAT<0.18 for each SEB. The analysis reiterated our results, and showed the levels of cryptic relatedness to be high in Tsonga and Pedi (~30 ±1.2 SD%), even after accounting for sample size differences (**Supplementary Fig. 10c**). Moreover, this analysis also demonstrates the cryptic-relatedness levels to be relatively high (range: 10-15%) in some of the other groups such as Swazi and Sotho (**Supplementary Fig. 10c**).

Note 5. Signatures of positive selection in SEB groups

We identified regions under positive selection in the SEB by estimating integrated haplotype scores (iHS) for each genic SNP in six SEB groups. All the SNPs that were observed to show extreme outliers iHS scores (|iHS|>4; *P*-value<0.003) in SEB populations are listed in **Supplementary Data 3**. **Fig. 4f** provides a comparison of the distribution of iHS scores for some of the genetic variants that are observed as outliers in at least two of the six SEB groups. As expected, the majority of these variants show uniformly high scores, although not always reaching the outlier threshold across all groups. However, for the outlier variants in genes such as *PAH, CAPN2,* and *SYT1,* the iHS were found to vary more widely between the six SEB groups (**Fig. 4f**). Although these variants emerged as outliers in some SEB groups, no evidence for selection was detected even at a relaxed *P*-value threshold of *P*<0.05 in other SEB groups. Moreover, as iHS can only be estimated for SNPs with a minimum MAF of 0.05, the allele frequencies of the outlier SNPs in genes such as *PPARG, RYR3,* and *SLC8A3* were found to be below this threshold in some of the SEB groups (shown by dark blue in the heatmap) (**Fig. 4f**).

To identify the possible functional impact of the signals, we classified the genes containing outlier SNPs according to ontology, pathway annotations and literature. The major functions represented by these genes include lipid metabolism, circadian regulation, response to oxygen levels, and immune related functions (**Fig. 4f).** One of these immune related genes, *LYAR,* has recently been shown to promote replication of multiple viruses such as influenza A virus (IAV), vesicular stomatitis virus (VSV), Japanese encephalitis virus (JEV), as well as to act as a negative regulator of innate immune responses[22].

Among known African selection signals, only *SYT1* (neurodevelopmental) and *FOXP2* (speech and language) were found to harbour an outlier SNP (|iHS|>4). However, we detected signatures of selection around other well-known selected regions, such as *LCT* (Lactase persistence), *LARGE* (Lassa fever), *OCA2* (skin pigmentation), and *VAV*3 (high altitude) at a moderate threshold of |iHS|>3 (*P*-value <0.05) (**Fig. 4g**). Interestingly, the signals in the *LCT* gene were found to reach moderate iHS (|iHS|>3) only in Xhosa. Moreover, the comparison of the difference in iHS values between SEB groups (**Supplementary Fig. 12)** shows the regions of difference to span a long genomic window (*LCT* in Xhosa) and even an entire gene (*GSK3B* in Tswana and Tsonga). A study (ref. [23]) has shown the presence of a variant in the *LCT* gene, that contributes to lactose persistence (LP) in appreciable frequencies in the Xhosa, and associated this with a high level of Khoe-San ancestry. For some of the well-known disease associated regions such as the DARC region, we did not detect any signature even at this moderate threshold. This could likely be due to the fact that the DARC region (in particular the Duffy-null allele) has been shown to have undergone a soft sweep instead of a hard sweep that is targeted by iHS based scans[24].

To identify variants showing high differentiation between SEB groups, we estimated population branch statistics (PBS) for variants between one pair of SEB groups and the Han Chinese population (CHB; ref. [12]) used as an outlier population (**Supplementary Table 9**). Genetic variants showing longer branch lengths (*P*-value <0.001) in Tswana, when compared to Tsonga, were detected within development related genes (*WLS)*, breast cancer associated genes (*BCSA3* and *BCSA4*) and a gene associated with ebola hemorrhagic fever (*NFKBIE*). The variants showing longer branch length in Tsonga when compared to Tswana, were found in key immune related genes (*VWF* and *ITGB2*), solute carrier genes (*SLC14A2, SLC35E3,* and *SLC1A1*), and the *MCHR1* gene, which play an important role in the control of feeding behaviour and energy metabolism (**Supplementary Table 9**).

Supplementary Tables

Supplementary Table 1. Description of datasets used in this study.

Dataset name	Sample size	Total SNPs	LD pruned	Description
AWI-S1	5,056	1,733,001	932,457	AWI-Gen-Set1 -All samples (1251 Pedi, 391 Sotho, 146 Swazi, 2110 Tsonga, 249 Tswana, 249 Venda, 178 Xhosa, and 656 Zulu individuals)
AWI-S2	4,319	1,733,001	932,457	AWI-Gen-Set2 - Unrelated samples only (PIHAT <0.18) (1065 Pedi, 366 Sotho, 126 Swazi, 1644 Tsonga, 242 Tswana, 73 Venda, 177 Xhosa, and 626 Zulu individuals)
AWI-S3	2,072	1,733,001	932,457	AWI-Gen-Set3- Unrelated and Ethnolinguistically concordant (EC) samples only (851 Pedi, 46 Sotho, 30 Swazi, 1438 Tsonga, 73 Tswana, 24 Venda, 63 Xhosa, and 177 Zulu individuals)
AWI-S4	476	1,733,001	932,457	AWI-Gen-Set4 - Unrelated and EC samples, some groups randomly downsized to obtain an homogenized sample size across groups (80 Pedi, 46 Sotho, 30 Swazi, 80 Tsonga, 73 Tswana, 24 Venda, 63 Xhosa, and 80 Zulu individuals)
Merged Dataset 1	5,426	1,259,001	800,261	AWI-S2 + selected populations from Gurdasani et al. (AGVP)[11], Auton et al. (KGP)[12], Schlebusch et al 2012[13]. Similar subsets for AWI-S3 and AWI-S4 were also generated.
Merged Dataset 2	5,631	723,218	416,372	LD-pruned AWI-S2+ Selected populations from Gurdasani et al. (AGVP), Auton et al. (KGP)[12], Schlebusch et al 2012[13], Choudhury et al. (SAHGP)[20], Semo et al.[25]. Similar subsets for AWI-S3 and AWI-S4 were also generated
AWI-AG	2,077	193,489	-	AWI-S3 + Ancient Genomes from Schlebusch et al.[26] and Skoglund et al.[27]
AWI-MV	934	25,647 X-chr	-	AWI-S3 + YRI & CEU from Auton et al. (KGP)[12] + Vicente et al.[28]

Supplementary Table 2. Comparison of ancestry proportions (inferred using ADMIXTURE at *K*=3) in South Eastern Bantu-speaking (SEB) groups sampled at the three study sites.

SEB Group	Bantu-speaker ancestry (%)		Khoe-San ancestry (%)		Eurasian ancestry (%)		Sample size
	Mean	±SD	Mean	±SD	Mean	±SD	
Pedi_AGT	93.45	5.13	6.18	5.24	0.36	0.38	33
Pedi_DKG	88.57	4.53	10.31	4.33	1.12	2.61	924
Pedi_SWT	84.22	7.04	14.46	5.36	1.32	4.50	108
Sotho_AGT	95.52	4.38	4.09	4.45	0.40	0.29	79
Sotho_DKG	88.47	4.43	10.70	5.01	0.83	0.91	9
Sotho_SWT	80.80	6.12	17.77	4.87	1.42	4.09	278
Swazi_AGT	94.97	5.71	4.12	4.91	0.91	3.23	70
Swazi_DKG	93.40	-	5.16	-	1.43	-	1
Swazi_SWT	84.61	6.77	14.58	6.03	0.81	1.49	55
Tsonga_AGT	98.18	1.70	1.23	1.38	0.59	0.99	1487
Tsonga_DKG	93.34	6.15	4.89	4.37	1.77	3.74	47
Tsonga_SWT	94.54	6.85	4.53	6.28	0.93	1.47	110
Tswana_AGT	78.57	-	21.36	-	0.08	-	1
Tswana_DKG	82.00	5.23	17.67	5.52	0.34	0.42	13
Tswana_SWT	77.97	7.52	20.65	6.04	1.38	4.72	228
Venda_AGT	96.87	2.25	1.96	1.74	1.17	0.53	5
Venda_DKG	92.02	5.75	5.41	3.19	2.57	5.39	21
Venda_SWT	90.41	7.57	7.40	5.77	2.20	3.78	47
Xhosa_AGT	78.12	1.01	20.54	0.34	1.34	0.84	3
Xhosa_DKG	81.68	5.15	16.54	5.06	1.77	0.68	6
Xhosa_SWT	80.23	5.98	17.61	4.90	2.16	3.25	168
Zulu_AGT	94.97	4.95	4.43	4.82	0.60	0.59	46
Zulu_DKG	83.92	5.36	14.59	3.38	1.49	2.51	8
Zulu_SWT	83.77	6.16	14.26	4.33	1.97	4.59	572

*AGT- Agincourt; DKG- Dikgale; SWT- Soweto

Supplementary Table 3. Timelines for Khoe-San (KS) and Bantu-speaker (BS) admixture in various South Eastern Bantu-speaking (SEB) groups estimated using three admixture dating methods: fastGLOBETROTTER, MALDER and MOSAIC.

		fastGLOBETROTTER		
**Target SEB group**	**Date in generations (±CI)**	**Date in years (±CI)**	**Inferred BS source (ancestry %)**	**Inferred KS source (ancestry %)**
Tsonga	44 (43-45)	645 (674-616)	Baganda (91.4%)	Karretjie (8.6%)
Venda	39 (38-40)	790 (819-761)	Baganda (89.1%)	Khomani (10.9%)
Pedi	33 (30-36)	964 (993-877)	Baganda (86.9%)	Karretjie (14.1%)
Sotho	28 (25-30)	1109 (1196-1051)	Baganda (74.4%)	Karretjie (25.6%)
Tswana	24 (21-27)	1225 (1312-1138)	Baganda (75.2%)	Karretjie (24.8%)
Swazi	31 (30-32)	1022 (1051-993)	Baganda (84.8%)	Karretjie (15.2%)
Zulu	31 (29-33)	1022 (1080-964)	Baganda (72.4%)	Karretjie (27.6%)
Xhosa	26 (24-28)	1167 (1225-1109)	Baganda (74.9%)	Karretjie (25.1%)

		MOSAIC		
**Target SEB group**	**Date in generations (±CI)**	**Date in years (±CI)**	**Inferred BS source (ancestry %)**	**Inferred KS source (ancestry %)**
Tsonga	40 (38-42)	761 (819-703)	Baganda (88.4%)	Khomani (11.6%)
Venda	29 (28-30)	1080 (1109-1051)	Baganda (91.3%)	Khomani (8.7%)
Pedi	30 (28-32)	1051 (1109-993)	Baganda (83.9%)	Karretjie (16.1%)
Sotho	26 (25-27)	1167 (1196-1138)	Baganda (78.2%)	Karretjie (21.8%)
Tswana	23 (20-26)	1254 (1341-1167)	Baganda (75.5%)	Karretjie (24.5%)
Swazi	25 (24-26)	1196 (1225-1167)	Baganda (86.6%)	Karretjie (13.4%)
Zulu	23 (22-24)	1254 (1283-1225)	Baganda (82.3%)	Karretjie (17.7%)
Xhosa	21 (20-22)	1312 (1341-1283)	Baganda (77.5%)	Karretjie (22.5%)

		MALDER		
**Target SEB group**	**Date in generations#**	**Date in years#**	**Inferred BS source**	**Inferred KS source**
Tsonga	45 (43-47)	616 (674-558)	Baganda	!Xun
Venda	45 (42-48)	616 (703-529)	Baganda	/Gui and //Gana
Pedi	29 (28-30)	1080 (1109-1051)	Baganda	Ju/'hoansi
Sotho	29 (28-30)	1080 (1109-1051)	Baganda	/Gui and //Gana
Tswana	26 (25-27)	1167 (1196-1138)	Baganda	!Xun
Swazi	29 (28-30)	1080 (1109-1051)	Baganda	Karretjie
Zulu	28 (27-29)	1109 (1138-1080)	Baganda	/Gui and //Gana
Xhosa	25 (24-26)	1196 (1225-1167)	Baganda	!Xun

*95% confidence intervals of estimates of dates and ancestry proportions estimated using fastGLOBETROTTER and MOSAIC were obtained by bootstrapping (see **Supplementary Notes 2**).

Values in bracket shows mean +/- standard error as provided by MALDER

All the generation times have been rounded

Supplementary Table 4. Timelines for Eurasian admixture in various* South Eastern Bantu-speaking (SEB) groups estimated using MALDER.

Target population	Source population 1 (Bantu-speaker)	Source population 2 (Eurasian)	Z	Date in generations#
Pedi	Baganda	CEU	13.56	4.78 +/- 0.35
Sotho	Baganda	CEU	13.53	3.67 +/- 0.27
Tsonga	Baganda	CEU	9.56	5.09 +/- 0.53
Tswana	Baganda	CEU	16.52	4.02 +/- 0.24
Venda	Baganda	CEU	7.86	3.26 +/- 0.42
Xhosa	Baganda	CEU	15.63	4.46 +/- 0.28
Zulu	Baganda	CEU	35.82	3.78 +/- 0.11

*Swazi had only 3 samples with more than 5% CEU ancestry and hence was excluded from the table
Time since admixture in generations (along with corresponding Z values) are displayed with standard errors provided by MALDER

Supplementary Table 5. Distribution of the five major Y-haplogroups in South Eastern Bantu-speaking (SEB) groups.

Y-haplogroups	Pedi	Tsonga	Tswana	Sotho	Swazi	Venda	Xhosa	Zulu	Total
A1b1b (M32)	11	14	6	9	4	0	5	6	55
B2b1 (M192)	2	31	0	1	1	1	0	2	38
B2a1a (M152)	54	133	33	24	11	9	7	30	301
E1b1 (P178)	260	480	80	105	51	17	72	221	1286
E2b (M98)	13	35	0	10	4	2	18	53	135
Total	340	693	119	149	71	29	102	312	1815

Supplementary Table 6. Mitochondrial haplogroup distribution in South Eastern Bantu-speaking (SEB) groups.

mtDNA-haplogroups	Pedi	Tsonga	Tswana	Sotho	Swazi	Venda	Xhosa	Zulu	Total
L0a2	22	48	6	6	2	1	3	14	102
L0a'b'f'g	2	0	2	0	0	0	1	1	6
L0a'b'g	196	314	44	57	25	17	33	94	780
L0d	2	1	0	0	0	0	0	1	4
L0d1	122	110	46	47	16	8	20	78	447
L0d2	108	57	32	47	7	8	27	80	366
L0d3	13	3	12	11	1	0	1	12	53
L0g	0	4	1	0	0	0	1	0	6
L0k	0	6	0	0	0	0	0	1	7
L1'2'3'4'5'6	35	80	1	15	10	4	7	33	185
L1b	4	8	0	1	0	2	0	7	22
L1c	59	118	7	20	6	4	5	28	247
L2	248	551	48	75	32	22	34	141	1151
L3d	44	67	3	21	10	3	9	30	187
L3e	112	207	31	52	13	4	25	89	533
L3f	3	23	2	1	3	0	2	1	35
L5	2	15	0	1	0	0	1	6	25
Other (M,N)	2	2	0	1	0	0	1	2	8
Total	974	1614	235	355	125	73	170	618	4164

Supplementary Table 7. *P*-value for differences in Khoe-San maternal ancestry contribution (based on comparison of X chromosome and autosomal contribution) between South Eastern Bantu-speaking (SEB) groups, estimated using Wilcoxon rank-sum (two tailed) test.

SEB Group	Pedi	Sotho	Swazi	Tsonga	Tswana	Venda	Xhosa
Sotho	2.5E-05						
Swazi	0.4242	2.0E-07					
Tsonga	0.0387	8.0E-09	0.0241				
Tswana	0.6993	1.6E-04	0.4758	0.0367			
Venda	0.0181	2.7E-05	7.8E-05	8.2E-10	0.0904		
Xhosa	0.0272	4.0E-03	8.7E-03	1.5E-05	0.2424	0.9223	
Zulu	0.3446	3.1E-06	0.5181	0.3197	0.2130	6.1E-04	1.3E-03

* *P*-values <0.01 are highlighted in grey.

Supplementary Table 8. Summary of association signals (mean and standard deviations) based on 50 simulated trait GWAS runs for each of the four categories and the respective subcategories, providing estimates for false positives due to population structure within South Eastern Bantu-speaking (SEB) groups.

Simulated sets	P-value		Mean	Std dev	Mean	Std dev	Mean	Std dev
			AGT_SWT		AGT_DKG		DKG_SWT	
Catagory 1: Simulates a scenario when cases and controls are sampled from different sites	Genome-wide hits	Unadjusted	21537.1	1116.2	4498.1	310.7	332.3	48.67
		PCA adjusted	0.06	0.24	0.12	0.43	0.14	0.35
		GC adjusted	0.08	0.44	0.28	0.57	0.08	0.27
		Genomic inflation	4.84	0.08	3.30	0.05	2.20	0.04
	Suggestive hits	Unadjusted	93517.0	3147.0	37888.8	1543.4	8820.6	682.8
		PCA adjusted	96.12	25.91	96.14	29.11	119.28	27.96
		GC adjusted	210.64	15.24	110.58	15.32	69.44	10.41
		Genomic inflation	4.84	0.08	3.30	0.05	2.20	0.04
			AGT(62.5%)_SWT(37.5%)		AGT(50%)_SWT(50%)		AGT(37.5%)_SWT(62.5%)	
Catagory 2: Simulates a scenario when cases are randomly drawn from two sites, and the controls are from one site only	Genome-wide hits	Unadjusted	1017.6	190.0	168.7	53.3	14.4	7.1
		PCA adjusted	0.10	0.36	0.10	0.46	0.08	0.27
		GC adjusted	0.02	0.14	0.04	0.20	0.10	0.36
		Genomic inflation	2.52	0.08	1.99	0.07	1.54	0.05
	Suggestive hits	Unadjusted	15858.6	1703.5	5873.4	990.1	1520.4	299.9
		PCA adjusted	83.48	14.18	78.50	12.71	78.10	12.82
		GC adjusted	87.16	16.55	73.10	12.54	64.60	10.70
		Genomic inflation	2.52	0.08	1.99	0.07	1.54	0.05
			SWT cases w/o Tswana		SWT cases w/o Tsonga			
Catagory 3: Simulates a scenario when both cases and controls are drawn from same site (SWT), but have unequal representation of SEB groups	Genome-wide hits	Unadjusted	0.68	1.03	0.08	0.27	-	-
		PCA adjusted	0.02	0.14	0.08	0.34	-	-
		GC adjusted	0.04	0.28	0.00	0.00	-	-
		Genomic inflation	1.18	0.03	1.11	0.02	-	-
	Suggestive hits	Unadjusted	258.30	58.38	155.84	25.67	-	-
		PCA adjusted	58.78	11.71	61.04	12.28	-	-
		GC adjusted	60.80	11.69	65.50	12.94	-	-
		Genomic inflation	1.18	0.03	1.11	0.02	-	-
			SWT Random		AGT Random		DKG Random	
Catagory 4: Simulates a scenario when we randomly assigned case and control status to individuals from same site	Genome-wide hits	Unadjusted	0.02	0.14	0.06	0.24	0.08	0.44
		PCA adjusted	0.02	0.14	0.04	0.20	0.08	0.44
		GC adjusted	0.02	0.14	0.06	0.24	0.08	0.44
		Genomic inflation	1.01	0.01	1.00	0.01	1.00	0.01
	Suggestive hits	Unadjusted	67.98	11.38	68.84	14.79	68.16	11.90
		PCA adjusted	55.64	10.25	56.08	12.65	54.28	9.76
		GC adjusted	65.14	11.54	66.42	12.99	65.44	11.47
		Genomic inflation	1.01	0.01	1.00	0.01	1.00	0.01

Unadjusted *P*-values correspond to GWAS association results derived by logistic regression (2-tailed).
Genomic inflation scores corresponding to these association test runs are shown.
Genome-wide hits were defined at the *P*-value threshold of $5x10^{-8}$ and suggestive hits at the *P*-value threshold of $1x10^{-5}$.
PC adjusted *P*-values correspond to association results with first 3 PCs as covariates.
GC adjusted *P*-values correspond to association results after genomic control based correction (using PLINK).
AGT- Agincourt; DKG- Dikgale; SWT- Soweto

Supplementary Table 9. Population Branch Statistics (PBS) outliers identified in Tswana-Tsonga-CHB and Tsonga-Tswana-CHB comparisons.

Tswana-Tsonga-CHB			
**Chromosome**	**Position**	**PBS score**	**Gene**
1	68695148	0.18802	*WLS*
1	68694377	0.15568	*WLS*
8	10876513	0.18297	*XKR6*
12	4671926	0.16848	*DYRK4*
20	49474361	0.16653	*BCAS4*
8	11285186	0.16166	*FAM167A*
8	11285186	0.16166	*C8orf12*
6	44232920	0.15425	*NFKBIE*
17	59374625	0.15063	*BCAS3*
14	69752603	0.14436	*GALNT16*
7	137139529	0.14135	*DGKI*

Tsonga-Tswana-CHB			
**Chromosome**	**Position**	**PBS score**	**Gene**
16	79625983	0.22705	*MAF*
2	26477650	0.22263	*HADHB*
4	160123686	0.20792	*RAPGEF2*
9	9804313	0.20353	*PTPRD*
22	41078473	0.19994	*MCHR1*
11	126372335	0.19617	*KIRREL3*
3	65753176	0.1887	*MAGI1*
12	6077639	0.16665	*VWF*
7	137004109	0.16122	*PTN*
6	162110497	0.15927	*PARK2*
1	159801155	0.15688	*SLAMF8*
3	1359249	0.15504	*CNTN6*
21	46311813	0.15419	*ITGB2*
18	43046905	0.15371	*SLC14A2*
8	30027098	0.15286	*LEPROTL1*
8	30027098	0.15286	*DCTN6*
1	3741985	0.14757	*CEP104*
10	6488790	0.14543	*PRKCQ*
4	5971082	0.14528	*C4orf50*
12	69179929	0.14372	*SLC35E3*
9	4513751	0.14307	*SLC1A1*
2	191371041	0.14219	*TMEM194B*
2	191371041	0.14219	*MFSD6*

Supplementary Figures

Supplementary Figure 1. Principal Component Analysis (PCA) based comparison of South Eastern Bantu-speaking (SEB) groups from the AWI-Gen study to previously studied populations from Southern and Eastern Africa, and 1000 Genomes Project Phase 3. a, PCA plot for SEB groups (Pedi N=851, Sotho N=46, Swazi N=30, Tsonga N=1438, Tswana N=73, Venda N=24, Xhosa N=63, Zulu N=177) are based on AWI-S3 dataset; Sotho_sahgp N=8 and Xhosa_sahgp N=7 (from ref. [20]); Zulu_agvp N= 99 and Sotho_agvp N=86 (from ref. [11]); Mozambique N=149 (from ref. [25]); SEBantu N=19 (from ref. [13]) and 3 Khoe-San (KS) groups /Gui //Ghana N=10, Ju|'hoansi N=14 and Karretjie N=17 (from ref. [13]). PC1 splits the KS from SEB groups while PC3 splits the geographically southern KS group (Karretjie people) from more northern KS groups as well as show the Sotho-Tswana speakers on one extreme and Nguni speakers on the other. **b**, PCA comparing SEB groups only, shows an overall concordance in localization of SEB groups from different datasets. For example, the Sotho from ref. [20] (Sotho_sahgp) and ref. [11] (Sotho_agvp) studies grouped together with AWI-Gen Sotho (Sotho). **c**, The PCA is based on SEB groups from AWI-S3 dataset and populations like Baganda N=96 from ref. [11]; ESN N=99, GWD N=113, LWK N=97, MSL N=85 and YRI N=108 from ref. [12]. As expected, on PC1 South African populations from our study split from Eastern and Western African populations from the 1000 Genomes Project Phase 3 (ref. [12]) and the AGVP (ref. [11]), while on PC2 the SEB samples with the highest Khoe-San admixture split from other SEB and African groups.

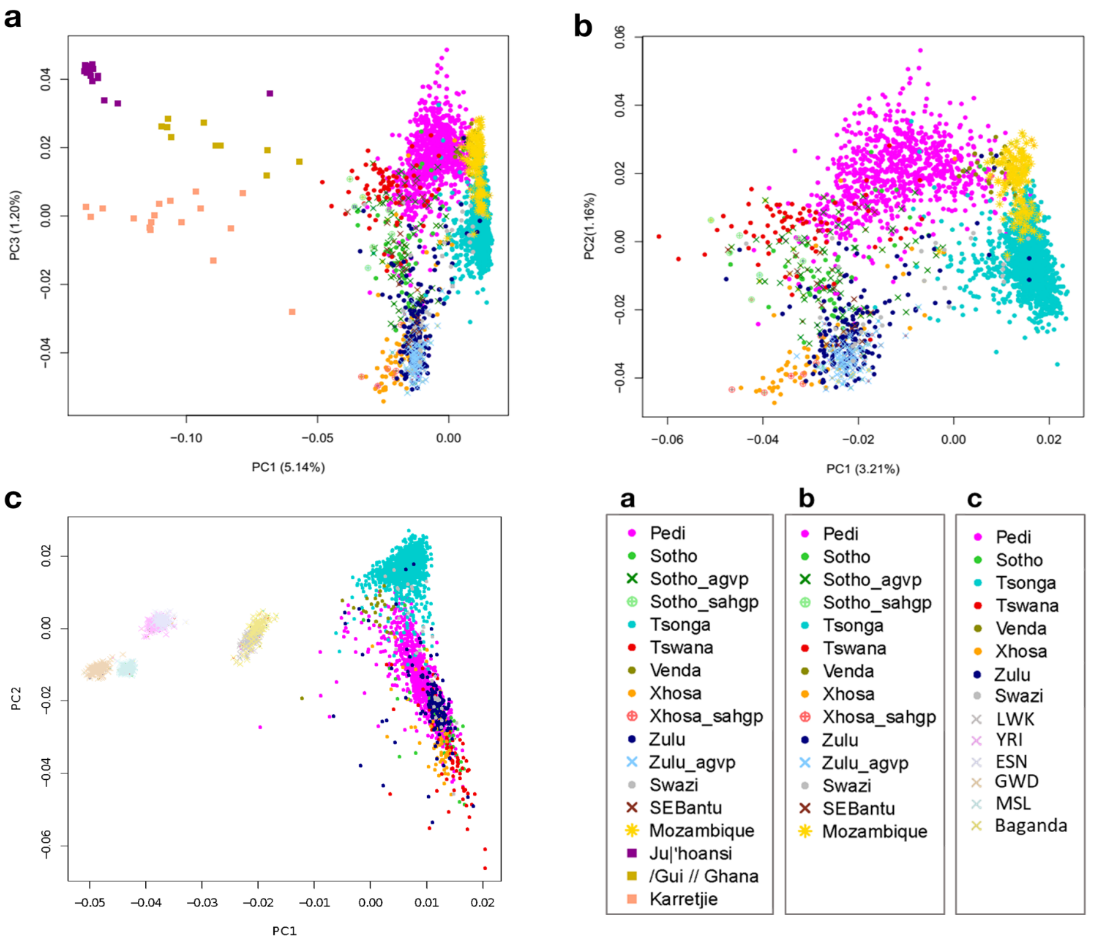

Supplementary Figure 2. Plots representing major South Eastern Bantu-speaking (SEB) groups included in this study downsized to a maximum of 80 ethno-linguistically concordant samples for each group. a, PCA plot showing PC1 and PC2. **b,** PCA-UMAP plot summarizing the composite of first 10 PCs. **c,** Haplotype-based PCA of SEB groups estimated on the basis of the co-ancestry matrix using fineSTRUCTURE. **d**, Average MCMC pairwise-coincidence matrix and tree estimated using fineSTRUCTURE clustering. Legend at the right represents the posterior coincidence probability, and SEB individuals at the left were colored using the same pattern of colors than in the PCA. Population labels highlight the major cluster identified among SEB groups. For **a-d**, the SEB groups (Pedi N=80, Sotho N=46, Swazi N=30, Tsonga N=80, Tswana N=73, Venda N=24, Xhosa N=63 and Zulu N=80) are based on AWI-S4 dataset.

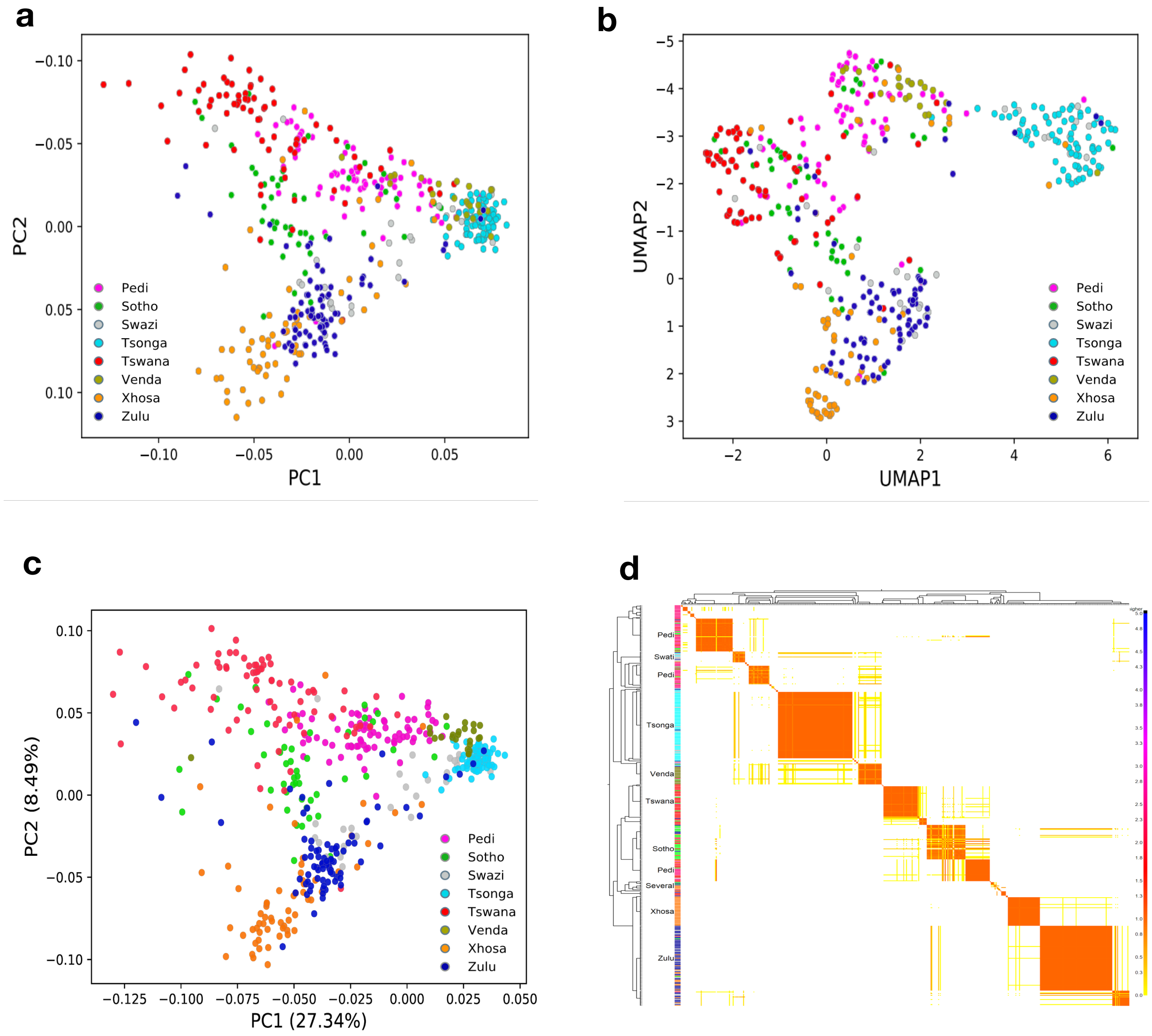

Supplementary Figure 3. Comparison of trees based on genetic and linguistic distances between the South Eastern Bantu-speaking (SEB) groups. a, UPGMA tree for pairwise F_{ST} distances between SEB groups from AWI-Gen study, ref. [11] (indicated by the suffix "_Agvp") and ref. [25] (named "Mozambique"). **b,** The full majority-rule consensus tree based on lexical data for 100 concepts in 69 Bantu language varieties, 34 of them part of South Eastern Bantu languages. **c,** Procrustes transformation analysis showing correlation (r^2=0.72; *P*-value=0.0009) between geographic distribution of SEB groups and rotated PCA plot on the South African map. The geometric midpoint of each SEB group is represented by its name in large font. PC location of individuals are shown using symbols in small fonts of same colour (that means z, Zulu; x, Xhosa; p, Pedi; t, Tswana; tg, Tsonga; s, Sotho; sz, Swazi; and ve, Venda). The *P*-values provided here are for the non-randomness ('significance') between geographic and genetic distribution matrices (two sided). **d,** Pairwise mean F_{ST} values between major SEB groups from the current study. For **a, c-d,** the data is based on SEB groups (Pedi N=80, Sotho N=46, Swazi N=30, Tsonga N=80, Tswana N=73, Venda N=24, Xhosa N=63 and Zulu N=80).

a

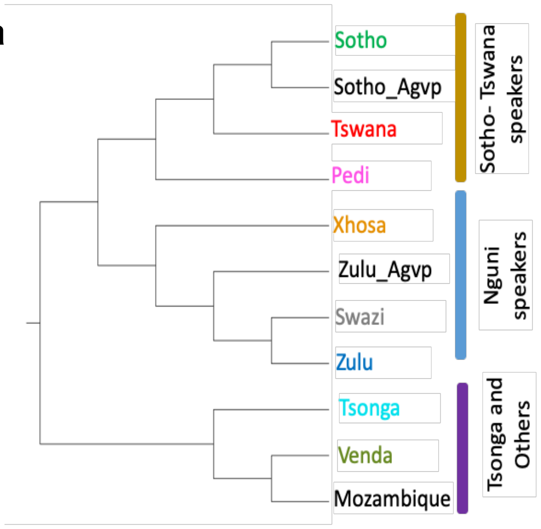

b

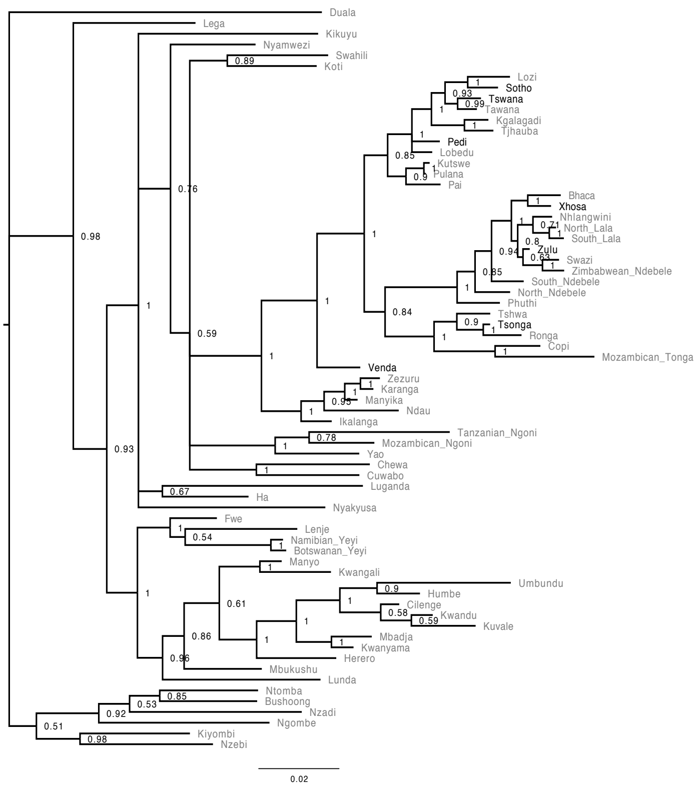

c

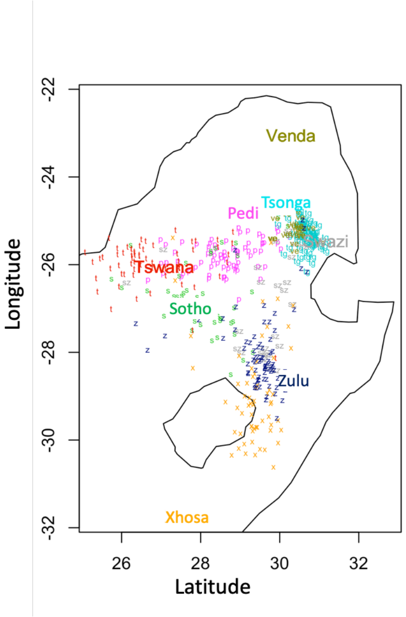

d

SEB group	Pedi	Sotho	Swazi	Tsonga	Tswana	Venda	Xhosa	Zulu
Pedi								
Sotho	0.0004							
Swazi	0.0006	0.0004						
Tsonga	0.0018	0.0024	0.0011					
Tswana	0.0011	0.0004	0.0021	0.0046				
Venda	0.0015	0.0016	0.0015	0.0011	0.0044			
Xhosa	0.0018	0.0007	0.0012	0.0039	0.0019	0.0032		
Zulu	0.0010	0.0005	0.0004	0.0026	0.0019	0.0027	0.0005	

Supplementary Figure 4. Geography strongly influences the levels of Khoe-San (KS) ancestry in some of the South Eastern Bantu-speaking (SEB) groups. a, Cross validation value plot for ADMIXTURE analysis between *K*=2 and *K*=8 (and ADMIXTURE results from *K*=3 to *K*=5 are shown in **Figure 2a**). **b**, ADMIXTURE plots at *K*=3 for a dataset with uniform representation of Eastern, Western and Southern African BS populations. **c**, ADMIXTURE plot at *K*=3 showing varying levels of Bantu-speaker like (blue), Khoe-San (green), and European-like (red) ancestry in all unrelated SEB individuals (N=4,319, AWI-S2). **d-f,** shows ADMIXTURE plots at *K*=3 for three of the SEB groups- (d) Zulu (e) Sotho (f) Swazi with sampling site information (AGT, Agincourt; DKG, Dikgale; and SWT, Soweto) appended to ethnolinguistic labels in the legend. **g,** *P*-values (based on two sided *t*-test) for differences in KS ancestry levels of SEB participants from the three sites. Comparisons showing *P*-values <0.05 are shown in green. Comparisons where there are no or very few samples in one of the sites are shown with "-".

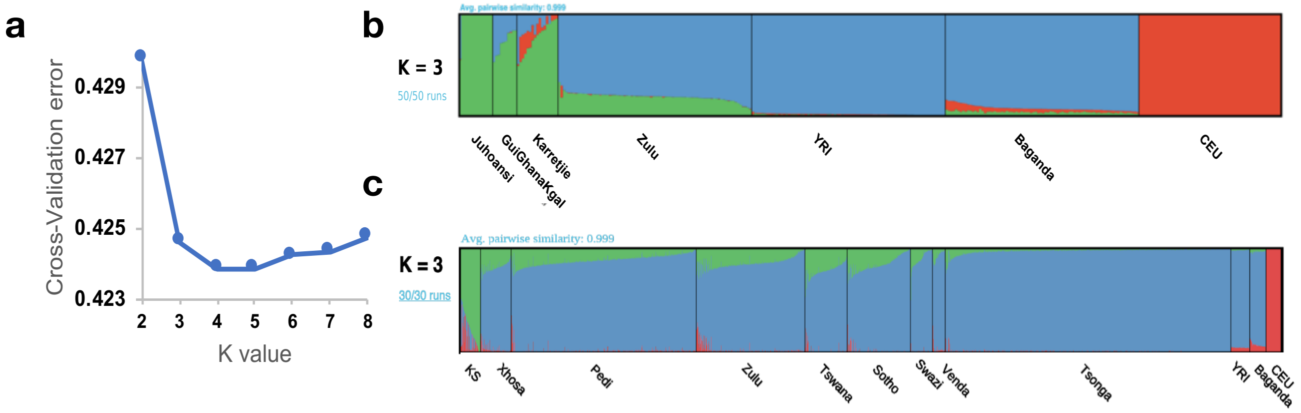

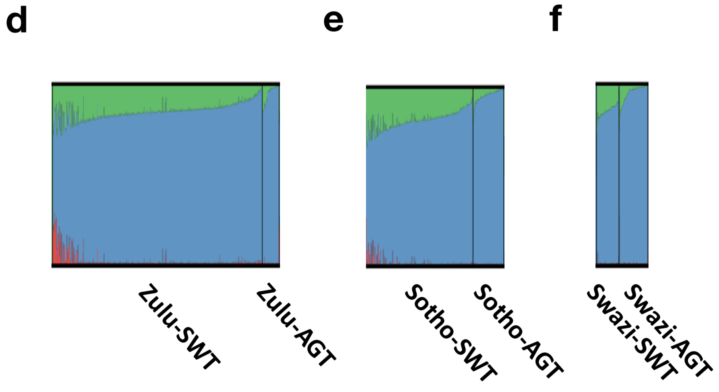

g

KS ancestry difference -P value

	AGT-SWT	AGT-DKG	DKG-SWT
Pedi	6.52E-13	5.80E-08	1.35E-19
Tsonga	1.29E-07	3.54E-07	3.39E-01
Sotho	2.13E-70	-	-
Swazi	1.45E-19	-	-
Venda	-	-	7.20E-02
Zulu	2.19E-42	-	-

Supplementary Figure 5. **Principal component analysis (PCA) plot showing South Eastern Bantu-speaking (SEB) groups from AWI-Gen labelled by both ethnicity and site (Soweto- SWT, Dikgale-DKG and Agincourt- AGT) of collection (and in parenthesis the sample size in each site).** In some cases the participants, instead of grouping together with other members of the same SEB group from another sampling site, tend to grouped with the participants of a different SEB group sharing the sampling-site. For example, some of the Zulu and Swazi participants sampled in AGT are closer to Tsonga (predominant group from AGT) rather than to participants from SWT or DKG, highlighting the importance of the site of sample collection.

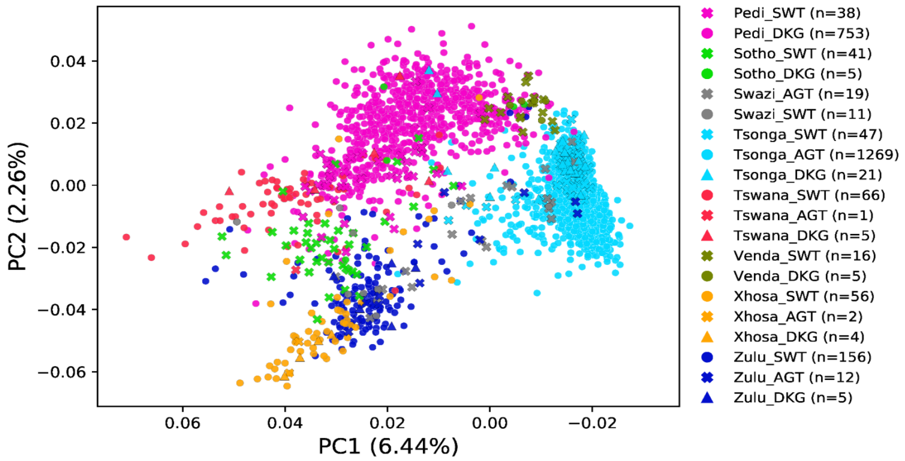

Supplementary Figure 6. Ancestry-specific principal component analysis plots showing persistence of population structure in South Eastern Bantu-speaking groups post Khoe-San ancestry masking using ancestry-specific principal component analysis approach. PCA plot is based on SEB groups Pedi N=851, Sotho N=46, Swazi N=30, Tsonga N=1438, Tswana N=73, Venda N=24, Xhosa N=63, Zulu N=177

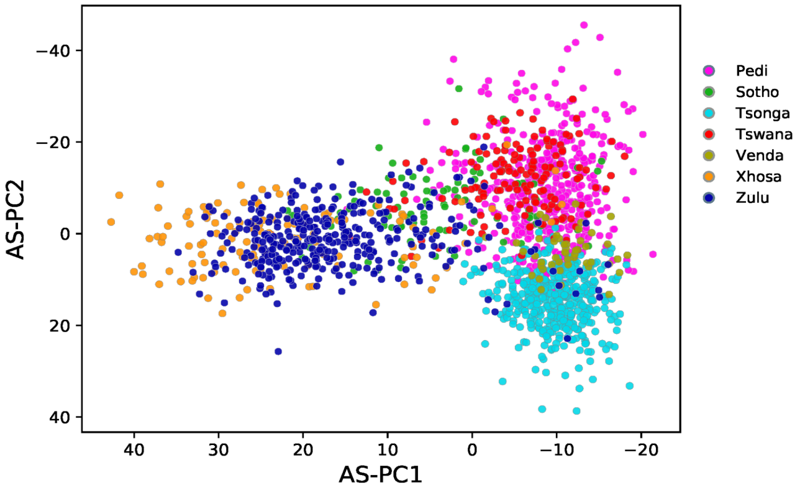

Supplementary Figure 7. Plot showing inferred two-way admixture dates (in generations) for each South Eastern Bantu-speaking (SEB) group estimated using fastGLOBETROTTER compared to dates estimated using a, MALDER and b, MOSAIC. Figure also showing 95% CI bars from MOSAIC and fastGLOBETROTTER estimates (obtained using bootstrapping) and standard errors for MALDER (obtained using chromosomal jack-knifing). Further details about these dates have been included in **Supplementary Table 3.**

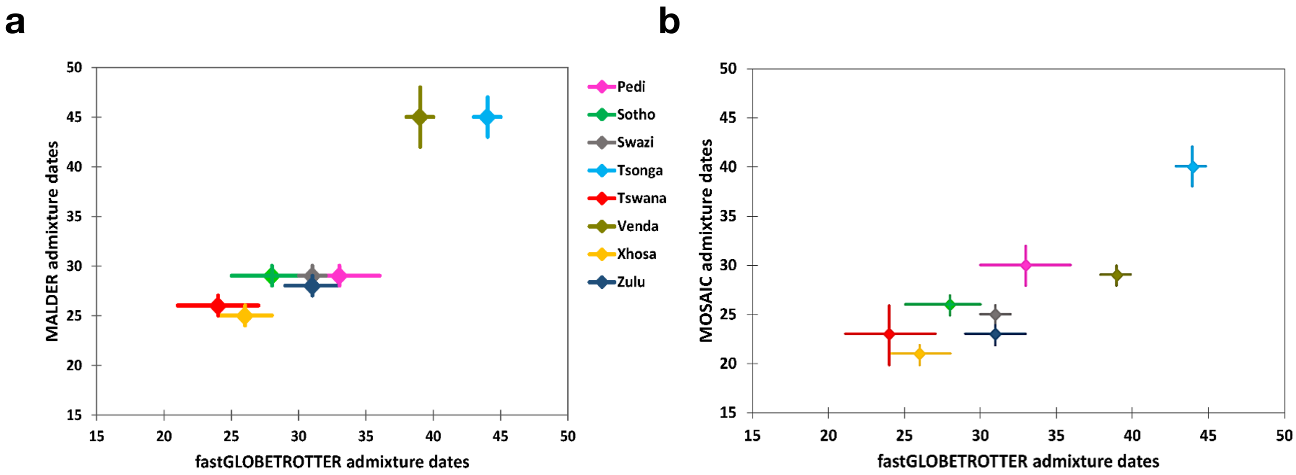

Supplementary Figure 8. Principal component analysis (PCA) plot showing Iron-Age genomes along with South Eastern Bantu-speaking (SEB) groups from the current study. PCA plot is based on SEB groups Pedi N=851, Sotho N=46, Swazi N=30, Tsonga N=1438, Tswana N=73, Venda N=24, Xhosa N=63, Zulu N=177 and selected ancient genomes from ref. [26] and ref [27].

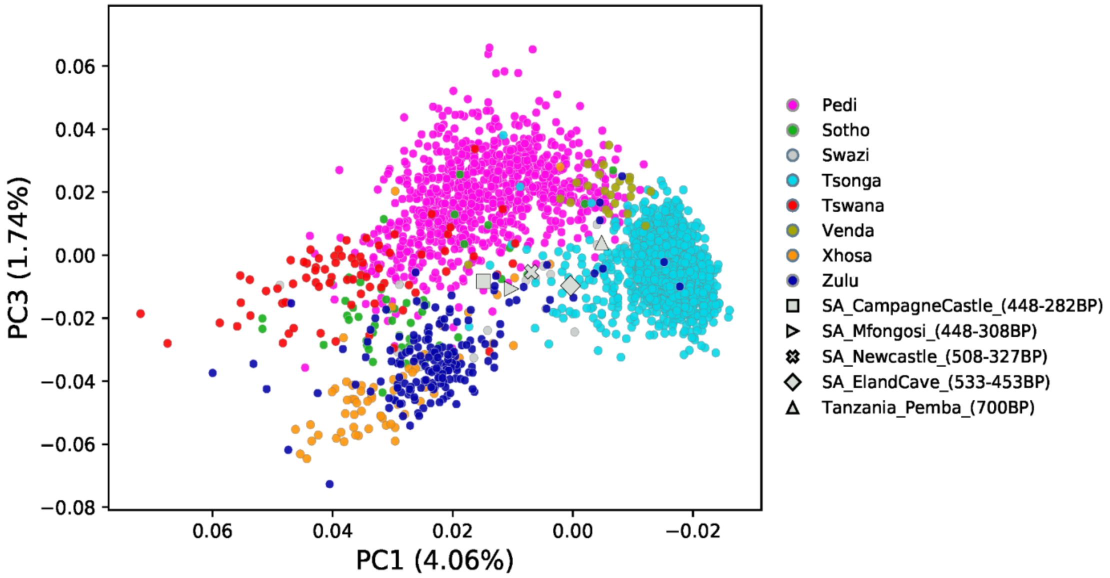

Supplementary Figure 9. **Comparison of effective population size (*Ne*) estimates for Sotho and Zulu from AGVP (ref. [11]) and the AWI-Gen study**. For both the datasets, Zulu maintain a lower *Ne* than Sotho around the period of ~15-40 generations. The similarity observed between the *Ne* profiles of Zulu AWI-Gen and Zulu_AGVP as well as Sotho AWI-Gen and Sotho-AGVP is despite the unequal sample size used for the analysis. For the AWI-Gen dataset, the sample size for both the groups was around ~220; while for the groups in the AGVP dataset the sample sizes were n=86 for Sotho and n=100 for Zulu. The shaded areas corresponding to each line demarcate 95% confidence intervals based on 80 bootstrapping runs.

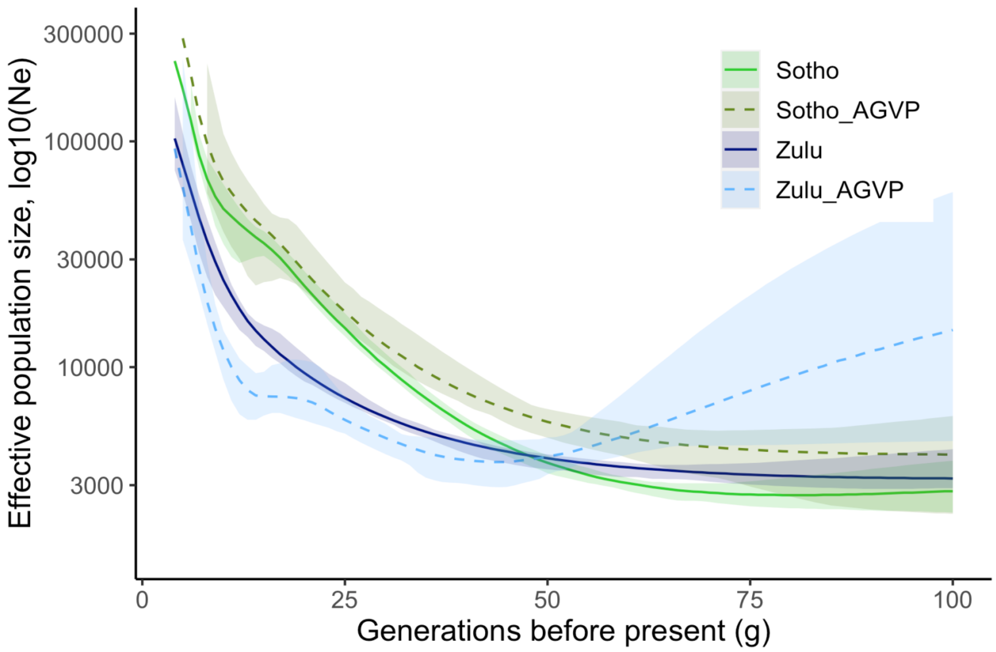

Supplementary Figure 10. **Relatedness levels in South Eastern Bantu-speaking (SEB) groups. a**, Relatedness at PIHAT>0.18 in groups stratified by study site **b**, Relatedness at 0.05<PIHAT<0.18 in groups stratified by study site **c**, Cryptic relatedness estimates (0.05<PIHAT<0.18) based on 100 resampling iterations consisting of up to 100 participants from each group (source data provided in Source Data file). The plots demonstrate very high levels of cryptic relatedness in Tsonga, sampled predominately from Agincourt (AGT), and Pedi, samples predominately from Dikgale (DKG).

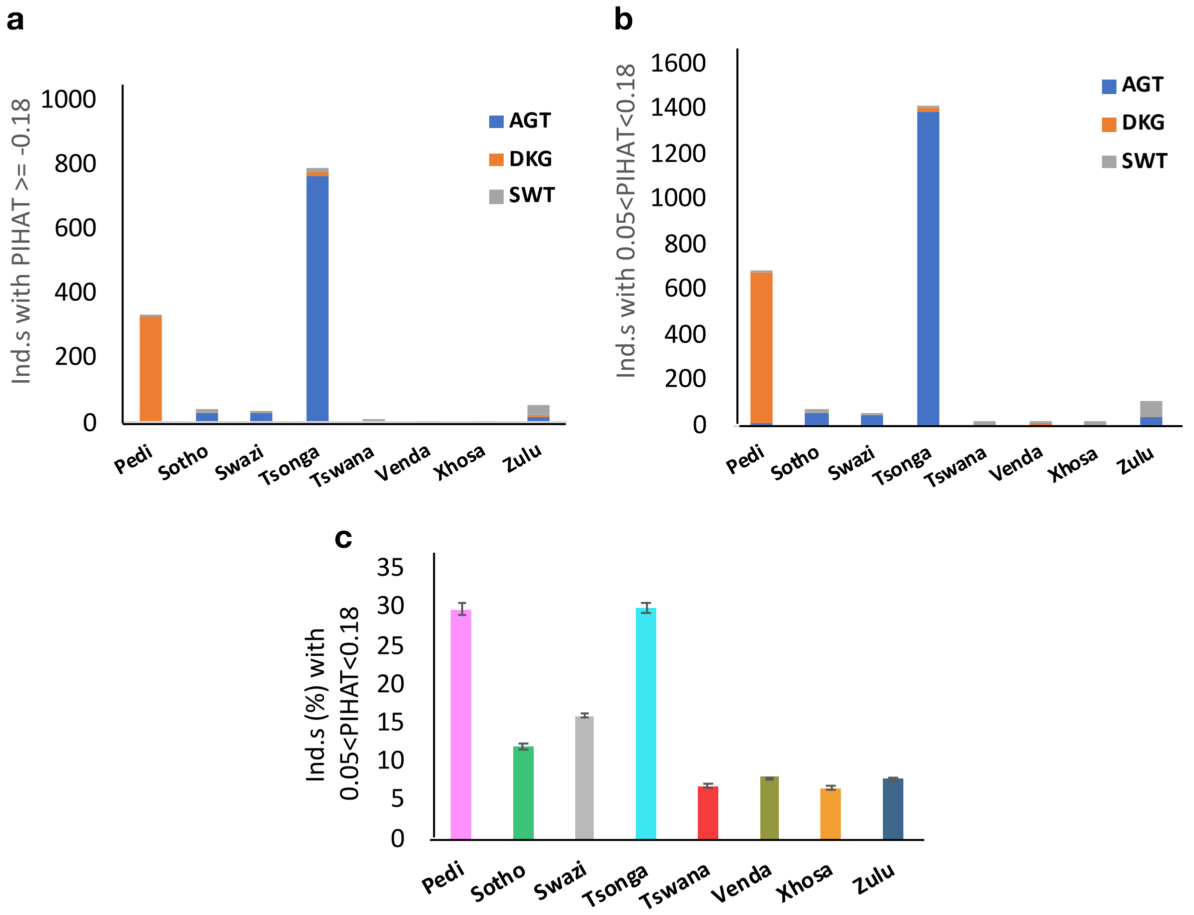

Supplementary Figure 11. Khoe-San (KS) and Bantu-speaking (BS) contributions to *Ne* profiles estimated using ancestry specific IBDNe in four South Eastern Bantu-speaking (SEB) groups: a, Sotho; b, Tsonga; c, Xhosa; and d, Zulu. The black line shows overall ("true") *Ne* while the red and blue lines show *Ne* for BS and KS components, respectively. The shaded areas corresponding to each line demarcate 95% confidence intervals based on 80 bootstrapping runs.

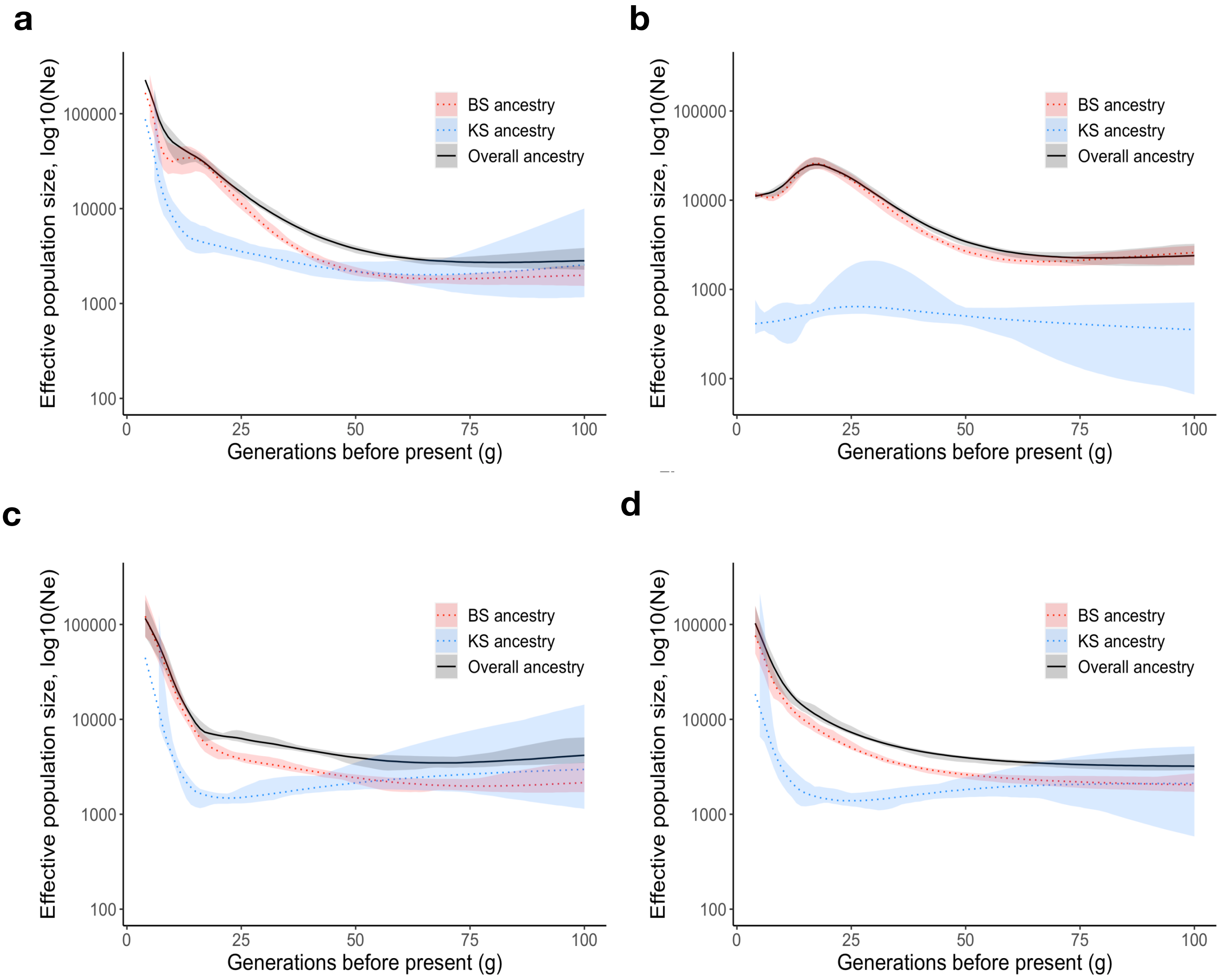

Supplementary Figure 12. Genomic regions showing high variation in iHS score distribution between South Eastern Bantu-speaking (SEB) groups. a, *LCT* and **b,** *GSK3B* genes**.** The x-axis in both (a) and (b) shows respective chromosomal coordinates.

a

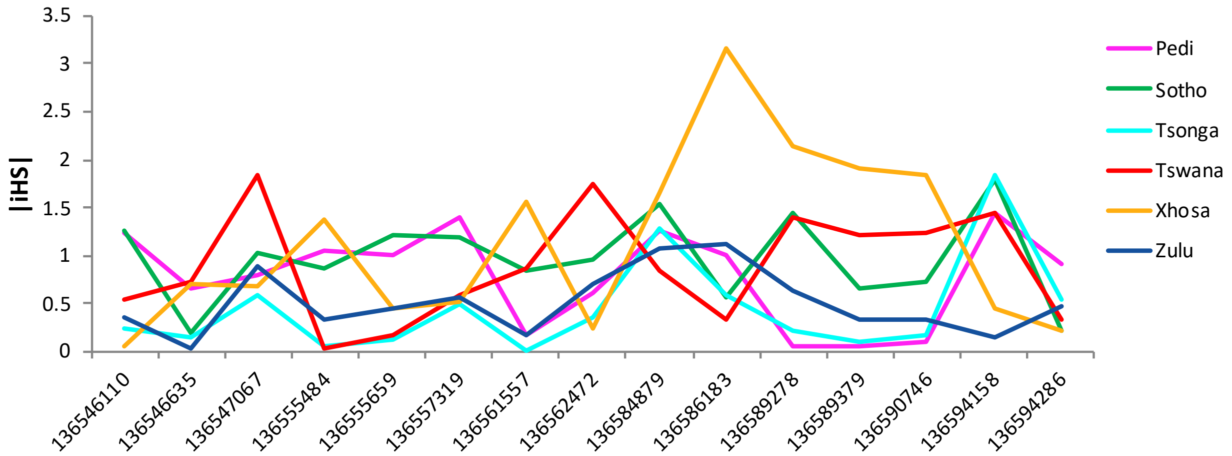

b

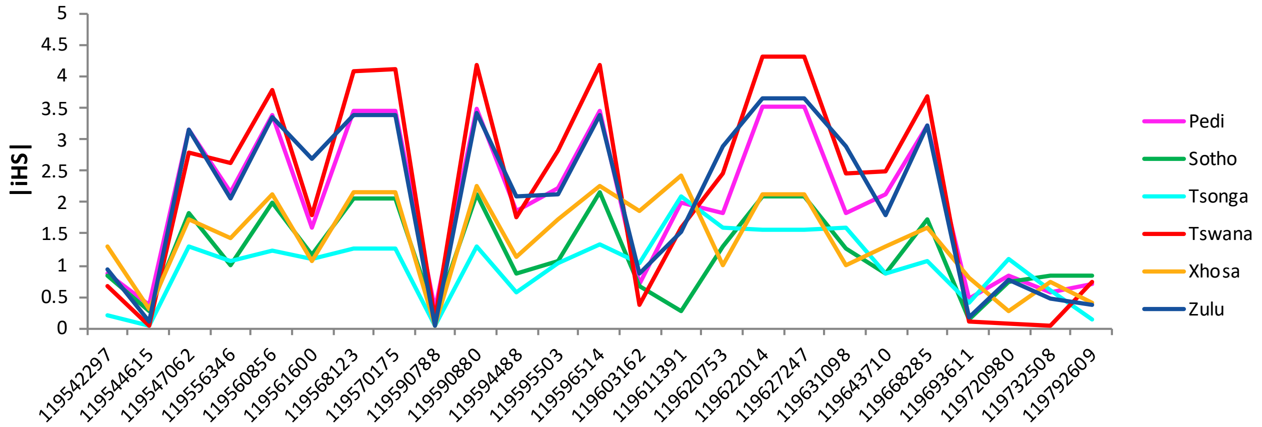

Supplementary Figure 13. Khoe-San (KS) local ancestry distribution across autosomal chromosomes in the South Eastern Bantu-speaking (SEB) group: a, Pedi; b, Sotho; c, Tswana; d, Zulu; e, Xhosa; f, Swazi; g, Tsonga; and h, Venda. The blue dotted lines represent Mean +/-3SD and the black dotted lines represent Mean +/-6SD.

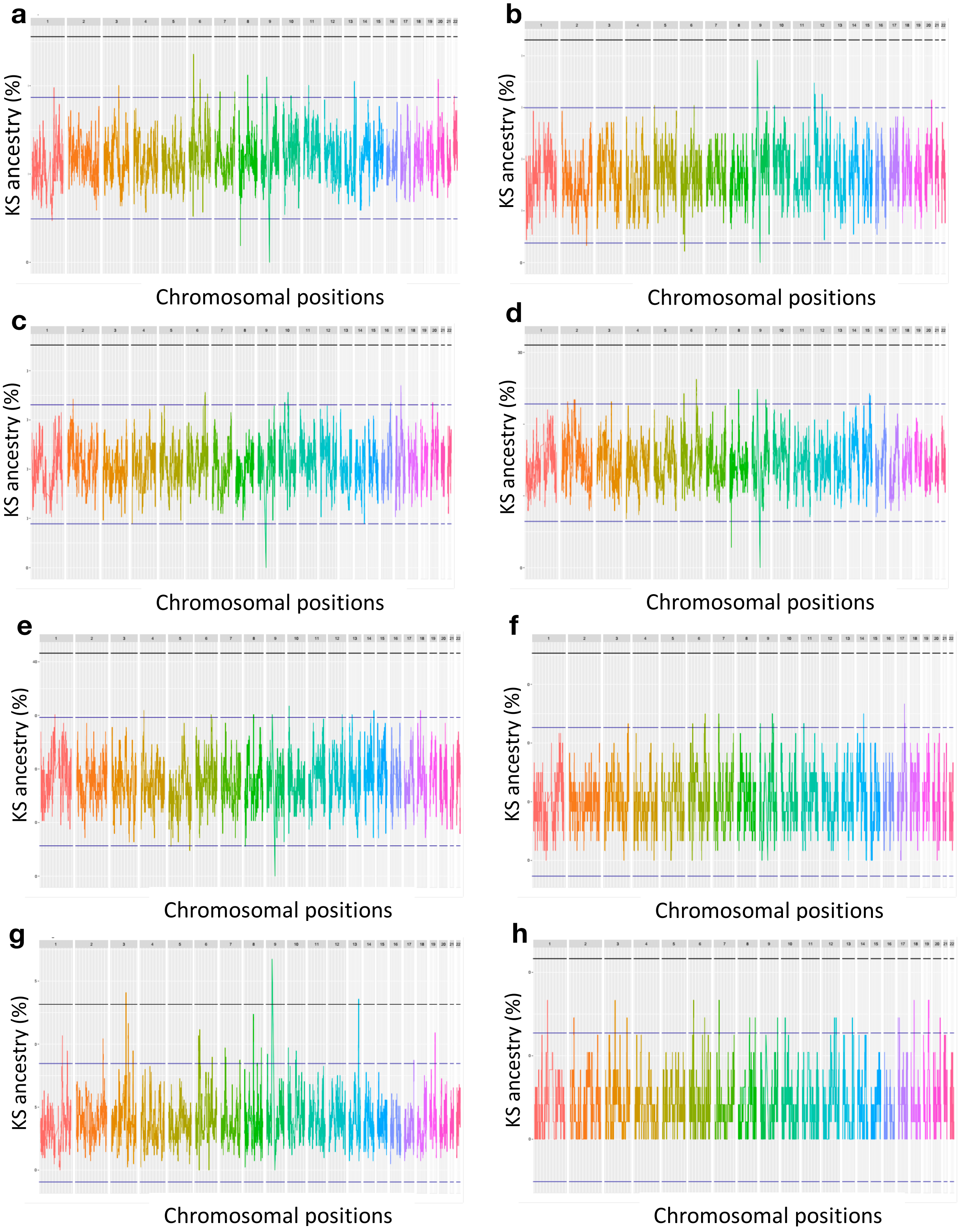

Supplementary Figure 14. PC-Relate plot depicting measures of pairwise genetic relatedness. Figure showing kinship coefficient (kin) and probability of sharing zero alleles IBD (k0), obtained using KING and GENESIS. According to the estimated kin and k0 values, low relatedness probabilities between pairwise AWI-Gen samples were found, therefore no first-degree or second-degree relatives were included in subsequent analyses.

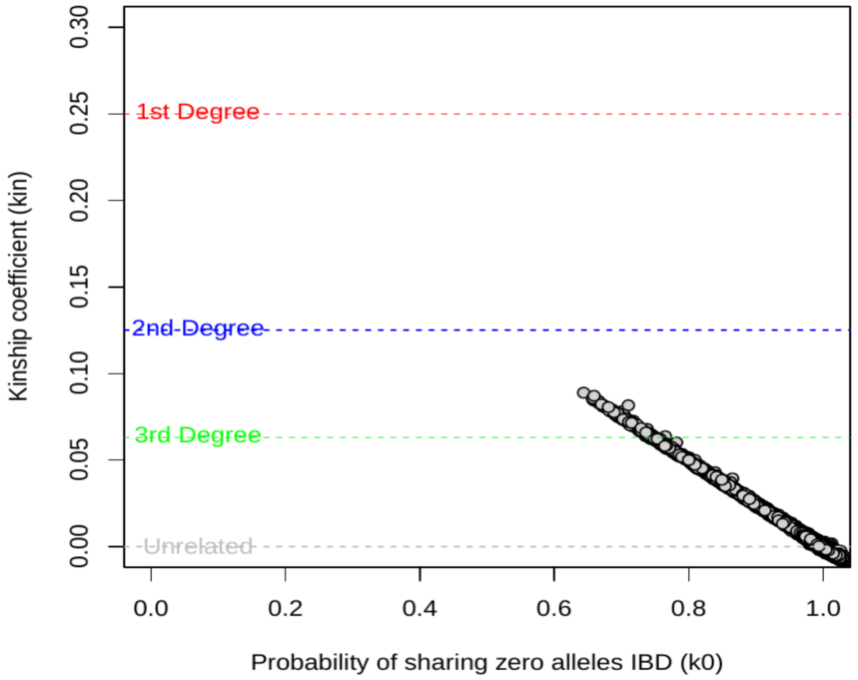

Supplementary Note References

1. Grollemund, R. *et al.* Bantu expansion shows that habitat alters the route and pace of human dispersals. *Proc. Natl. Acad. Sci. U. S. A.* **112**, 13296–13301 (2015).
2. Ronquist, F. & Huelsenbeck, J. P. MrBayes 3: Bayesian phylogenetic inference under mixed models. *Bioinformatics* **19**, 1572–1574 (2003).
3. Huelsenbeck, J. P. & Ronquist, F. MRBAYES: Bayesian inference of phylogenetic trees. *Bioinformatics* **17**, 754–755 (2001).
4. Felsenstein, J. Phylogenies from restriction sites: a maximum-likelihood approach. *Evolution* **46**, 159–173 (1992).
5. Loubser, J.H.N. The ethnoarchaeology of Venda-speakers in southern Africa. *Navorsinge van die Nasionale Museum Bloemfontein* **7** (8), 146–464 (1991).
6. Huffman, T.N. *Handbook to the Iron Age: The Archaeology of Pre-Colonial Farming Societies in Southern Africa*. (University of KwaZulu-Natal Press, 2007).
7. Pakendorf, B., Gunnink, H., Sands, B. & Bostoen, K. Prehistoric Bantu-Khoisan language contact: A cross-disciplinary approach. *Language Dynamics and Change* **7**, 1–46 (2017).
8. Lawson, D. J., Hellenthal, G., Myers, S. & Falush, D. Inference of population structure using dense haplotype data. *PLoS Genet.* **8**, e1002453 (2012).
9. Li, N. & Stephens, M. Modeling linkage disequilibrium and identifying recombination hotspots using single-nucleotide polymorphism data. *Genetics* **165**, 2213–2233 (2003).
10. Hellenthal, G. *et al.* A genetic atlas of human admixture history. *Science* **343**, 747–751 (2014).
11. Gurdasani, D. *et al.* The African Genome Variation Project shapes medical genetics in Africa. *Nature* **517**, 327–332 (2015).
12. 1000 Genomes Project Consortium *et al.* A global reference for human genetic variation. *Nature* **526**, 68–74 (2015).
13. Schlebusch, C. M. *et al.* Genomic variation in seven Khoe-San groups reveals adaptation and complex African history. *Science* **338**, 374–379 (2012).
14. Loh, P.-R. *et al.* Inferring admixture histories of human populations using linkage disequilibrium. *Genetics* **193**, 1233–1254 (2013).
15. Salter-Townshend, M. & Myers, S. Fine-Scale Inference of Ancestry Segments Without Prior Knowledge of Admixing Groups. *Genetics* **212**, 869–889 (2019).
16. Price, A. L. *et al.* Sensitive detection of chromosomal segments of distinct ancestry in admixed populations. *PLoS Genet.* **5**, e1000519 (2009).
17. Fenner, J. N. Cross-cultural estimation of the human generation interval for use in genetics-based population divergence studies. *Am. J. Phys. Anthropol.* **128**, 415–423 (2005).
18. Bajić, V. *et al.* Genetic structure and sex-biased gene flow in the history of southern African populations. *Am. J. Phys. Anthropol.* **167**, 656–671 (2018).
19. Schlebusch, C. M. Genetic variation in Khoisan-speaking populations from southern Africa. (University of the Witwatersrand Johannesburg (South Africa, 2010).
20. Choudhury, A. *et al.* Whole-genome sequencing for an enhanced understanding of genetic variation among South Africans. *Nat. Commun.* **8**, 2062 (2017).
21. Chang, C. C. *et al.* Second-generation PLINK: rising to the challenge of larger and richer datasets. *Gigascience* **4**, 7 (2015).
22. Fang, Y. *et al.* New strains of Japanese encephalitis virus circulating in Shanghai, China after a ten-year hiatus in local mosquito surveillance. *Parasit. Vectors* **12**, 22 (2019).
23. Ranciaro, A. *et al*. Genetic origins of lactase persistence and the spread of pastoralism in Africa. *Am. J. Hum. Genet.* **94**, 496–510 (2014).
24. McManus, K. F. *et al.* Population genetic analysis of the DARC locus (Duffy) reveals adaptation from standing variation associated with malaria resistance in humans. *PLoS Genet.* **13**, e1006560 (2017).
25. Semo, A. *et al.* Along the Indian Ocean Coast: Genomic Variation in Mozambique Provides New Insights into the Bantu Expansion. *Mol. Biol. Evol.* **37**, 406–416 (2020).
26. Schlebusch, C. M. *et al.* Southern African ancient genomes estimate modern human divergence to 350,000 to 260,000 years ago. *Science* **358**, 652–655 (2017).

27. Skoglund, P. *et al.* Reconstructing Prehistoric African Population Structure. *Cell* **171**, 59–71.e21 (2017).
28. Vicente, M., Jakobsson, M., Ebbesen, P. & Schlebusch, C. M. Genetic Affinities among Southern Africa Hunter-Gatherers and the Impact of Admixing Farmer and Herder Populations. *Mol. Biol. Evol.* **36**, 1849–1861 (2019).
