# Supplementary material for: Genetic substructure and complex demographic history of South African Bantu speakers
Source: Nat Commun. 2021 Apr 7;12:2080. doi: 10.1038/s41467-021-22207-y (PMC8027885; doi:10.1038/s41467-021-22207-y)
Supplement: Supplementary file 3 — Description of Additional Supplementary Files [file 41467_2021_22207_MOESM3_ESM.pdf]

Description of Additional Supplementary Files

File Name: Supplementary Data 1.

Description: Phenotype associated variants (inferred using GWAS catalog and categorized by disease/trait) showing at least 3 fold allele frequency difference between at least two South Eastern Bantu-speaking (SEB) groups. Columns 2-7 are based on information from the GWAS catalog (accessed on 19th April, 2020). Columns 10 to 15 show allele frequencies of each variant in SEB groups. "Min'' and "Max" indicate the highest and lowest frequency observed, respectively. "Fold" shows the ratio of maximum to minimum frequency. Associations detected in GWASs that include African ancestry populations in the discovery cohort are shown in blue.

File Name: Supplementary Data 2.

Description: Known phenotype associated SNPs (according to GWAS catalog) that were identified as signals at the genome wide significant threshold P-value < 5X10-8 (association results derived by logistic regression (2-tailed) using PLINK) in at least ten of the simulated trait GWAS iterations

File Name: Supplementary Data 3.

Description: Comparison of absolute iHS scores in six South Eastern Bantu-speaking (SEB) groups showing differences in signal strengths. Corresponding gene names for SNVs showing outlier scores in at least one of the SEB groups are shown.

File Name: Supplementary Data 4.

Description: Genomic regions showing outlier (>mean+3SD) KhoeSan (KS) ancestry in the 6 South Eastern Bantu-speaking (SEB) groups.
