# Supplementary material for: Genetic substructure and complex demographic history of South African Bantu speakers
Source: Nat Commun. 2021 Apr 7;12:2080. doi: 10.1038/s41467-021-22207-y (PMC8027885; doi:10.1038/s41467-021-22207-y)
Supplement: Supplementary file 8 — Reporting Summary [file 41467_2021_22207_MOESM8_ESM.pdf]

nature research

Corresponding author(s): Michele Ramsay

Last updated by author(s): Dec 20, 2020

Reporting Summary

Nature Research wishes to improve the reproducibility of the work that we publish. This form provides structure for consistency and transparency in reporting. For further information on Nature Research policies, see our Editorial Policies and the Editorial Policy Checklist.

Statistics

For all statistical analyses, confirm that the following items are present in the figure legend, table legend, main text, or Methods section.

n/a	Confirmed	
☐	☒	The exact sample size (n) for each experimental group/condition, given as a discrete number and unit of measurement
☒	☐	A statement on whether measurements were taken from distinct samples or whether the same sample was measured repeatedly
☐	☒	The statistical test(s) used AND whether they are one- or two-sided<br>*Only common tests should be described solely by name; describe more complex techniques in the Methods section.*
☐	☒	A description of all covariates tested
☐	☒	A description of any assumptions or corrections, such as tests of normality and adjustment for multiple comparisons
☐	☒	A full description of the statistical parameters including central tendency (e.g. means) or other basic estimates (e.g. regression coefficient) AND variation (e.g. standard deviation) or associated estimates of uncertainty (e.g. confidence intervals)
☐	☒	For null hypothesis testing, the test statistic (e.g. F, t, r) with confidence intervals, effect sizes, degrees of freedom and P value noted<br>*Give P values as exact values whenever suitable.*
☒	☐	For Bayesian analysis, information on the choice of priors and Markov chain Monte Carlo settings
☒	☐	For hierarchical and complex designs, identification of the appropriate level for tests and full reporting of outcomes
☒	☐	Estimates of effect sizes (e.g. Cohen's d, Pearson's r), indicating how they were calculated

Our web collection on statistics for biologists contains articles on many of the points above.

Software and code

Policy information about availability of computer code

Data collection	No software was used for data collection.
Data analysis	PLINK 1.9, VCFtools 0.1.15, EIGENSOFT 7.2.1, UMAP, ADMIXTURE 1.3.0, pong v1.4.9, fastGLOBETROTTER, MALDER 1.0, MOSAIC 1.3.7, BEAGLE-5, SHAPEIT-2, IBD-Ne (ibdne.07May18.6a4.jar), ASIBD-Ne pipeline (https://faculty.washington.edu/sguy/asibdne/posted_commands.txt), KING 1.4, GENESIS 2.10.1, Selscan v1.1.0b, MEGA X 10.0.4, RFMix-1.54, AMY-tools, SNAPPY_v0.1, Haplogrep-2, ASPCA pipeline (http://faculty.washington.edu/sguy/local_ancestry_pipeline/rfmix_mds_pipeline), In-house plotY tool (https:/github.com/shaze/ymthaplotools), MrBayes v3.2.7, Python library umap-learn 0.3.7, R packages (MCMCpack v1.4-9, vegan v2.5-6, Gmedian v1.2.5)

For manuscripts utilizing custom algorithms or software that are central to the research but not yet described in published literature, software must be made available to editors and reviewers. We strongly encourage code deposition in a community repository (e.g. GitHub). See the Nature Research guidelines for submitting code & software for further information.

Data

Policy information about availability of data

All manuscripts must include a data availability statement. This statement should provide the following information, where applicable:

- Accession codes, unique identifiers, or web links for publicly available datasets
- A list of figures that have associated raw data
- A description of any restrictions on data availability

Genotype data from the AWI-Gen study have been deposited in the European Genome-phenome Archive (EGA; https://ega-archive.org/) with the accession number: EGAD00010001996 [https://www.ebi.ac.uk/ega/datasets/EGAD00010001996]. DNA samples are archived in H3Africa biorepositories as part of the H3Africa Consortium agreement. The Data and biospecimens are available to interested researchers through EGA, subject to controlled access review by the Data and Biospecimen Access Committee of the H3Africa Consortium.

April 2020

Publicly available datasets included in the study are the following:
1000 Genomes Project (ftp://ftp.1000genomes.ebi.ac.uk/vol1/ftp/),
SAHGP (https://www.ebi.ac.uk/ega/studies/EGAS00001002639),
Schlebusch et al, 2012 (http://jakobssonlab.iob.uu.se/data/),
AGVP (https://www.ebi.ac.uk/ega/studies/EGAS00001000960),
Vicente et al., 2019 (https://www.ebi.ac.uk/arrayexpress/experiments/E-MTAB-7813/),
Semo et al., 2019 (https://www.ebi.ac.uk/arrayexpress/experiments/E-MTAB-8450/),
Schlebusch et al. 2017 (https://www.ebi.ac.uk/ena/browser/view/PRJEB22660)
Skoglund et al 2017 (https://www.ebi.ac.uk/ena/browser/view/PRJEB21878)

Field-specific reporting

Please select the one below that is the best fit for your research. If you are not sure, read the appropriate sections before making your selection.

- [x] Life sciences
- [ ] Behavioural & social sciences
- [ ] Ecological, evolutionary & environmental sciences

For a reference copy of the document with all sections, see nature.com/documents/nr-reporting-summary-flat.pdf

Life sciences study design

All studies must disclose on these points even when the disclosure is negative.

Sample size	The AWI-Gen cohort consists of more than 5000 South African participants, recruited between 2013 and 2016 at three study sites in the country. To maximize the representation of each South-Eastern Bantu-speaker group as well as genetic diversity in country we included all the participants from this cohort in our study. This makes this study one of the largest population genetic study preformed in continental African populations. However, for specific analyses such as PCA, admixture and selection, subsets of participants from each SEB group were randomly sampled to maintain uniformity of sample sizes.
Data exclusions	Exclusions was based on standard criteria such as genotype quality (sample missingness and SNP missingness, deviation from Hardy-Weinberg equilibrium, minor allele frequency) and relatedness between samples.<br>Only the QC-ed data was used for all the experiments. The only exception was the analysis of relatedness, which for obvious reasons included the related samples
Replication	For analyses such as as PCA, admixture and population size dynamics, we performed a joint analysis of our data with previously generated independent datasets for the same SEB groups (if available) and compared the clustering (for PCA), ancestry composition (for ADMIXTURE) and effective population size dynamics of participants. In all these cases we observed high concordance between the trends observed in our data and the independent datasets.
Randomization	In our study the groupings are primarily based on self-reported ethnolinguistic identity Therefore, covariates or controlling for covariates are not relevant to our study.
Blinding	The study design did not allocate samples to specific groups such as "cases" or "controls" and compare them. Instead, we have investigated levels of genetic differences between and within self-reported ethno-linguistic groups. Moreover, for some of the analyses such as PCA and ADMIXTURE, even the ethno-linguistic information was projected on to the analysis results and did not predetermine/influence the outcomes.

Reporting for specific materials, systems and methods

We require information from authors about some types of materials, experimental systems and methods used in many studies. Here, indicate whether each material, system or method listed is relevant to your study. If you are not sure if a list item applies to your research, read the appropriate section before selecting a response.

Materials & experimental systems

n/a	Involved in the study	
x		Antibodies
x		Eukaryotic cell lines
x		Palaeontology and archaeology
x		Animals and other organisms
	x	Human research participants
x		Clinical data
x		Dual use research of concern

Methods

n/a	Involved in the study	
x		ChIP-seq
x		Flow cytometry
x		MRI-based neuroimaging

Human research participants

Policy information about studies involving human research participants

Population characteristics	Individuals were chosen to represent the ethno-linguistic diversity of South Eastern Bantu-speakers from South Africa. The participants are population cross section based with no intended enrichment of any trait/diseases. In addition to ethno-linguistic identity of the participants, self-reported ethno-linguistic identity of the parents and grandparents, if available, were also recorded. The participants included both males and females, predominantly in the age range of 40-60 years. The cohort is population cross sectional and did not include or exclude participants based on trait, disease or ethno-linguistic identity.
Recruitment	A total of 5268 participants were recruited from three existing cohorts -Agincourt (AGT), Dikgale (DKG) and Soweto (SWT) in Mpumalanga, Limpopo and Gauteng provinces of South Africa, respectively, under the Africa-Wits-INDEPTH partnership for genomic studies (AWI-Gen) project as part of the Human Heredity and Health In Africa (H3Africa) Consortium. The recruitment was preceded by community engagement. The participation was completely voluntary and there were no advantages or disadvantages for opting in or opting out of the study respectively. Given that the aim of our study was primarily to assess the background genetic diversity at each of the study sites we do not anticipate the recruitment process to have introduced major biases. However, for some of the SEB groups (such as Xhosa), none of the study sites, correspond to the linguistic majority areas, and therefore the representation of these groups in our dataset might be sub-optimal.
Ethics oversight	This study was approved by the Human Research Ethics Committee (Medical) of the University of the Witwatersrand (Wits) (protocol number M121029), and renewed in 2017 (protocol number M170880). In addition, research at the Dikgale Study Centre was approved by the Medunsa Research and Ethics Committee of the University of Limpopo (MREC/HS/195/2014:CR). Community engagement preceded sample collection and all participants provided broad consent for medical and population genetic studies.

Note that full information on the approval of the study protocol must also be provided in the manuscript.
